# Editorial: Selective brain and heart hypothermia - A path toward targeted organ resuscitation and protection

**DOI:** 10.3389/fneur.2023.1162865

**Published:** 2023-03-14

**Authors:** Jae H. Choi, John Pile-Spellman, Judah Weinberger, Sven Poli

**Affiliations:** ^1^Neurovascular Center, NSPC Brain and Spine Surgery, Lake Success, NY, United States; ^2^Dean's Office, Touro University, NYSCAS, New York, NY, United States; ^3^Department of Neurology and Stroke, University of Tübingen, Tübingen, Germany; ^4^Hertie Institute for Clinical Brain Research, University Hospital and Faculty of Medicine, University of Tübingen, Tübingen, Germany

**Keywords:** neuroprotection, therapeutic hypothermia, selective brain hypothermia, selective cardiac hypothermia, targeted organ resuscitation, precision medicine, acute ischemic stroke, intracerebral hemorrhage

## Stroke—When survival is not all that matters

Recently, remarkable improvements in outcomes have been made possible for patients with acute brain ischemia (1–5). With a rate of successful reperfusion in over 80% of cases using mechanical thrombectomy, a good outcome can now be achieved in as many as half of patients with large vessel occlusion-related stroke, while the death rate is less than 20% (6). However, “good outcome” does not mean “back to life as it was before the stroke.” In fact, “excellent outcome” is found only in less than one-fifth of patients, which highlights the severe nature of the condition and the urgent need for additional treatment to protect the brain.

## Hypothermia—When a threat becomes a cure

Cardiac arrest is the most severe form of ischemia, where due to a total collapse of cardiac activity whole body ischemia ensues. Typically, <10% of patients survive cardiac arrest, a prognosis that is far grimmer than for stroke (7). Interestingly, observations have revealed that the survival rate could increase to as high as 50% when cardiac arrest coincided with *accidental deep hypothermia* (e.g., accidents in snowy mountains or drowning in icy waters), a critical condition where the body core temperature drops to 28°C or below (8). Here, hypothermia produces a state of resistance to ischemic cell death through mechanisms that are tissue-protective and especially, neuroprotective (9, 10). In fact, systematic studies have confirmed a clinical benefit of post-ischemic mild therapeutic hypothermia (TH) in neonates with ischemic encephalopathy and found the relevance of hypothermia in patients following cardiac arrest and during cardiac surgery (11–13).

Unsurprisingly, preclinical experiments in stroke models were conclusive about the neuroprotective properties of hypothermia (14). However, the results from clinical trials of systemic TH in acute ischemic stroke patients were more sobering and showed that it was simply not a trivial task to induce systemic hypothermia in awake patients, even if the degree of hypothermia was only mild (33–34°C) (15, 16). In this Research Topic, Hong et al. summarize the clinical challenges and complications that typically occur during the phases of systemic hypothermia in acute stroke patients, i.e., phases of induction, maintenance, and re-warming, a lengthy procedure that may last up to 72 h. The challenges are serious and caused by natural mechanisms to fight core cooling, complications of prolonged body hypothermia, and adverse rebound phenomena.

## Therapeutic hypothermia—From systemic cooling to selective brain hypothermia

Still, are there reasons why we should remain enthusiastic about TH? Perhaps it is the mechanism of hypothermic cellular protection. In their review article, You et al. state that TH “may be the most robust neuroprotective modality in laboratory and preclinical studies.” The pleiotropic mechanisms of protection affect multiple physiological systems, such as metabolism, blood flow, excitotoxicity, cell death pathways, and inflammation.

Or perhaps it is what we can learn from nature, from hibernating animals that can manipulate body temperature to extreme conditions to withstand periods of low energy and oxygen supply. Having extensively studied arctic ground squirrels in their natural habitat in Alaska and also in the laboratory, Drew et al. contribute a review work on the complex physiology of hibernation torpor. Some of the insights may hold the key to success for patients suffering from cerebral ischemia.

Then how could hypothermic neuroprotection be harnessed to be applied more safely in acute stroke patients? Targeted temperature management with maintaining normothermia and fighting fever has been suggested as a safer approach instead of core hypothermia (Baker et al.; You et al.). Another possibility to exploit the true potential of neuroprotective hypothermia is selective brain hypothermia, a concept that would provide targeted cerebral cooling and avoid the dangers of systemic hypothermia. Speed and depth of brain tissue hypothermia are obvious advantages. Hong et al. offer a review of the concepts of systemic and selective brain hypothermia and provide a detailed summary of the different cooling methods. In this regard, Wang et al. contribute with an updated review of preclinical experiments and clinical studies on selective brain cooling in acute cerebral ischemia.

Although less frequent, intracerebral and intraventricular hemorrhages are important clinical conditions where additional neuroprotection is needed. Baker et al. have put together a Consensus Recommendation paper on TH in intracerebral hemorrhage patients who undergo minimally invasive hematoma evacuation. Recommended TH modalities include systemic or selective brain cooling and combinations thereof. In addition, the authors suggest hypothermia protocols that should be useful in future clinical investigations in patients with intracerebral and intraventricular hemorrhages.

Mattingly et al. present their findings from an experiment on selective brain hypothermia in a pig stroke model using a sophisticated extracorporeal cooling and bypass concept. The demonstrated non-linear relationship between depth and duration of hypothermia and stroke volume suggests that there might be an optimal “dose” of selective brain hypothermia.

Lastly, Horn et al. review alternative non-invasive methods to monitor brain temperature that could be applied during selective organ cooling procedures. The methods include magnetic resonance spectroscopy (MRS), radiometric thermometry, and microwave radiometry.

## Selective brain and heart hypothermia—A path toward targeted organ resuscitation and protection

Recognized as being extremely challenging to safely achieve systemic hypothermia in awake stroke patients, novel *selective brain cooling* technologies are currently under development, and early clinical investigations have been encouraging. Similarly, *selective endovascular cardiac cooling* for cardioprotection has been studied in patients with acute myocardial ischemia. Although initial clinical results have been promising (17, 18), it is unlikely that the translation of selective brain or cardiac hypothermia could be widely successful without the proper technology, as both organs are embedded in the body and shielded well against thermal stress (19). However, there is little doubt about the tissue-protective mechanisms of hypothermia, which are robust across species, including humans.

The Editors of this Research Topic applaud the authors for their contributions to a young clinical discipline and targeted organ treatment paradigm. Here, selective brain hypothermia could pave the path toward targeted organ resuscitation and protection (20).

## Author contributions

JC and SP have drafted and finalized the editorial. JP-S and JW have reviewed and revised the draft. All authors contributed to the article and approved the submitted version.
